# Natural Compounds in the Battle against Microorganisms—Linalool

**DOI:** 10.3390/molecules27206928

**Published:** 2022-10-15

**Authors:** Wanda Mączka, Anna Duda-Madej, Małgorzata Grabarczyk, Katarzyna Wińska

**Affiliations:** 1 Department of Food Chemistry and Biocatalysis, Wrocław University of Environmental and Life Sciences, Norwida 25, 50-375 Wrocław, Poland; 2 Department of Microbiology, Wroclaw Medical University, Chałubińskiego 4, 50-368 Wrocław, Poland

**Keywords:** linalool, monoterpene, antimicrobial, biotransformation

## Abstract

The purpose of this article is to present recent studies on the antimicrobial properties of linalool, the mechanism of action on cells and detoxification processes. The current trend of employing compounds present in essential oils to support antibiotic therapy is becoming increasingly popular. Naturally occurring monoterpene constituents of essential oils are undergoing detailed studies to understand their detailed effects on the human body, both independently and in doses correlated with currently used pharmaceuticals. One such compound is linalool, which is commonly found in many herbs and is used to flavor black tea. This compound is an excellent fragrance additive for cosmetics, enhancing the preservative effect of the formulations used in them or acting as an anti-inflammatory on mild skin lesions. Previous studies have shown that it is extremely important due to its broad spectrum of biological activities, i.e., antioxidant, anti-inflammatory, anticancer, cardioprotective and antimicrobial. Among opportunistic hospital strains, it is most active against Gram-negative bacteria. The mechanism of action of linalool against microorganisms is still under intensive investigation. One of the key aspects of linalool research is biotransformation, through which its susceptibility to detoxification processes is determined.

## 1. Introduction

Linalool (C_10_H_18_O, 3,7-dimethyl-1,6-octadien-3-ol) is a monoterpene acyclic tertiary alcohol with a global production market valued at USD 9980 million in 2019 and expected to reach USD 12,300 million in 2024 [1]. It is mainly obtained by chemical synthesis [2]. As a fragrance compound, it has found numerous applications in the production of perfumes, lotions, soaps, and shampoos as well as in non-cosmetic household products such as detergents and cleaners. An interesting option in recent years is the possibility of using linalool as an active packaging ingredient, for example, by injecting it into the space above the contents of food packaging or even by applying it to food coatings and films. The problem of its high volatility and effect on the contents of the package can be solved by encapsulating it in micro- or nano-emulsions [3]. In turn, in the pharmaceutical industry, it is used as a precursor for the synthesis of vitamin E [4]. Linalool is the most widely used monoterpene in food as its presence has been reported in 23 foods, albeit in varying amounts. In orange and bergamot juices, the content of linalool was 6740–34,430 µg/L and in apricots, grapes, nectarines, peaches, strawberries, and tomatoes, it ranged from less than 10 µg/kg to over 600 µg/kg, while in lemon, melon, and raspberries, it was less than 10 µg/kg. It is worth noting that the addition of jasmine to green tea significantly increases the content of linalool in the range from 30,500 to even 1,315,000 µg/kg [5]. Linalool and its acetate are the main flavors in Earl Gray teas [6]. Linalool is also found in many other plants, being a key component of their volatile fractions. Table 1 contains an overview of plants with a linalool content of more than 10% in the volatile fraction.

Linalool represents about 70% of the floral fragrances [20] and exists as two enantiomers: (3*S*)-(+)-linalool (coriandrol) and (3*R*)-(−)-linalool (licareol). It is worth noting that both enantiomers have different fragrance profiles: coriandrol is perceived as sweet, floral, herbal and petitgrain-like with citrus and fruity notes, while licareol has a woody, lavender aroma. The odor threshold of the (3*R*)-(−)-enantiomer is approximately nine times lower than that of the (3*S*)-(+)-linalool (0.8 vs. 7.4 ppb). It may also exist in racemic form (e.g., passion fruit and apricots). The racemate is also a product of the fermentation processes involved in the production of foodstuffs. In plants, linalool is made from geranyl pyrophosphate (GPP)—a universal monoterpene precursor, which is produced by the condensation of isopentenyl pyrophosphate (IPP) and dimethylallyl diphosphate (DMAPP) by GPP synthase [21]. It may also be a byproduct of the biosynthetic pathways of other terpenoids, e.g., geraniol and nerol. Linalool accumulates in the compartmentalized secretory structures of glandular hairs or is directly emitted into the environment [22]. Thanks to the tools of molecular biology, linalool can also be produced by microorganisms. *Escherichia coli* strains were able to produce (3*R*)-(−)-linalool by overproducing its precursor, mevalonate. On the other hand, the use of bacterial linalool/nerolidol synthase (bLinS) from *Streptomyces clavuligerus* allowed linalool to be obtained in an amount of up to 500 mg/L. However, bLinS also accepts farnesyl diphosphate as a substrate that is naturally produced by *E. coli*; therefore, *trans*-nerolidol was formed as a by-product (~30% of the total product mix), making this technology difficult for commercial applications [4]. The (3*S*)-(+)-linalool synthase from *Actinidia arguta* was in turn introduced into *Saccharomyces cerevisiae* and *Yarrowia lipolytica*. In contrast, in *Synechocystis* spp. PCC 6803 cyanobacteria, the co-expression of the above (3*S*)-(+)-linalool synthase and *E. coli* farnesyl pyrophosphate synthase mutant S80F acting as GPP synthase was used. In the case of *Pantoea ananatis*, the solubility of the same (3*S*)-(+)-linalool synthase was improved by the N-terminal fusion of the halophilic β-lactamase from *Chromohalobacter* spp. 560 with hexahistidine [23]. Since linalool is cytotoxic to microorganisms, the instability of the DNA pathway constructs and the maintenance of a fully functioning metabolic pathway are major problems. For this reason, it is imperative to minimize genetic recombination that leads to loss of enzyme(s)/pathway. One can also try to increase the host microbial tolerance to linalool using adaptive laboratory evolution tools to limit the selection of mutant strains that do not have a fully functional pathway during fermentation [4].

Linalool exhibits a number of other biological activities in addition to its antimicrobial activity, which will be discussed in detail in this publication. Thanks to its anti-inflammatory [24,25] and antioxidant [26] properties, administration of linalool to mice protected them against peripheral inflammation caused by exposure to the endotoxin *Salmonella* Typhimurium [27]. Linalool also increased anti-inflammatory and antioxidant markers and restored kidney integrity in a rat model of streptozotocin-induced diabetic nephropathy [28]. Linalool also inhibits LDL oxidation and enhances cholesterol uptake by macrophage scavenger receptors. Linalool significantly lowered the levels of plasma triglycerides, total cholesterol and HMG-CoA, demonstrating antiatherosclerotic activity in vivo [29]. (3*R*)-(−)-Linalool can interact with nitric oxide synthase (NOS) to inhibit nitric oxide production without reducing enzyme synthesis. NO regulates inflammatory and immune responses, contributing to the formation of edema, vasodilation, and the recruitment of immune cells at the site of infection [30]. It is presumed that the anti-inflammatory activity of linalool is responsible for its neuroprotective effects in rats with hemiparkinsonism. It significantly prevented the downregulation of tyrosine hydroxylase and dopamine transporter in a rat model of Parkinson’s disease [31]. Linalool also reduced TNF-α-induced inflammation in brain-derived endothelial cells [32]. It also inhibited the pro-inflammatory pathways induced by LPS and the production of cytokines (e.g., nitric oxide, NF-κB, TNF-α, IL-6 and IL-1β) in murine microglia (BV2) cells [33,34]. In the triple transgenic model of Alzheimer’s disease (3xTg-AD) mice, 21–24 months of age, the influence of linalool (25 mg/kg) on the development of this disease was investigated. The 3xTg-AD mice administered linalool showed better learning and spatial memory, and better risk assessment behavior during an elevated plus maze. In addition, a significant reduction in extracellular β-amyloidosis, tauopathy, astrogliosis and microgliosis in the hippocampus and amygdala was found, as well as a significant reduction in the level of pro-inflammatory markers p38 MAPK, NOS2, COX2 and IL-1β. On this basis, researchers suggest that linalool may play an important role in preventing Alzheimer’s disease [34,35].

In addition to its anti-inflammatory and antioxidant properties, linalool also exhibits anti-cancer [36,37,38], sedative, anxiolytic and anticonvulsant effects. The effectiveness of dietary supplements containing (3*R*)-(−)-linalool in the treatment of anxiety disorders has been confirmed by clinical trials [39].

During inhalation, maximum transfer of linalool to the brain occurs after 90 min. Inhalation with racemic linalool restored the expression level of many stress-induced genes to normal. These genes were associated with synaptic transmission mediated by neurotransmitters, including anxiolytic neuropeptides such as oxytocin and neuropeptide Y, and contained MHC class I proteins that were required for proper brain neurodevelopment and plasticity. Additionally, Yoshida et al. suggested that inhalation of linalool may activate prolactin [40]. However, researchers believe that further pharmacokinetic and pharmacodynamic studies are needed to understand the influence of linalool and its metabolites on the course of nerve signals, especially in clinical neuropathologies [34].

In turn, Mohamed et al. [41] investigated the cardioprotective effect of linalool on isoproterenol-induced myocardial infarction in rat models. Linalool reduced the size of the infarct area and escalated HO-1 and Nrf2, both nuclear and cytosolic fractions, and decreased Keap 1. Linalool abolished the apoptotic response and enhanced the antioxidant activity of the heart by increasing the level of Bcl2 and by reducing the level of TNF-α, NF-κB cytokines, IL-1β, and IL-6, as well as markers of apoptosis (caspase-3, caspase-9 and *Bax*).

Linalool also has an effect on vascular smooth muscle cells (VSMC). Long-term hypertension leads to changes in the structure and function of blood vessels, and abnormal VSMC proliferation and migration are important factors in these changes. Liang et al. [42] investigated the basics of the mechanism of action of linalool and its influence on the physiological behavior of VSMC. In VSMC studies, angiotensin II (Ang II) was used. Linalool inhibited Ang II-induced proliferation and migration of VSMCs and blocked the MAPK signaling pathway by downregulating the muscarinic cholinergic receptor 3 (CHRM3).

The main purpose of the review is to present recent studies on the antimicrobial properties of linalool. Since every compound introduced into the organism undergoes transformations in it, the first part discusses the metabolism of linalool, with a special focus on the reactions of this compound catalyzed by CYP. In turn, when discussing the antimicrobial properties of linalool, we focused on potential mechanisms that may be responsible for this activity of linalool. New in our review is a detailed review of the microbial biotransformation pathways that linalool may undergo in the environment. Now more than ever, it is important to be aware of the human impact on the environment, and it is important not to contribute to increased pollution when bringing new products to market.

## 2. Metabolism of Linalool

Although linalool has several beneficial effects on the human body, little data are available on its metabolism in humans. After inhalation for 45 min (10% *v/v* (3*R*)-(−)-linalool in propylene glycol solution), the compound appeared in the blood after 25 min and lasted for about 40–45 min [43]. On the other hand, when (3*R*)-(−)-linalool in peanut oil was applied to the skin, terpene alcohol was detected in the blood about 30 min after the massage. Interestingly, when applied to the skin, males showed better absorption than females. The longer time of penetration into the blood after application to the skin may be due to its accumulation in the subcutaneous fatty tissue [44]. Linalool readily penetrates the stratum corneum and the rate of decline is estimated at 10–20% per hour after cessation of exposure [45]. In the case of massage using lavender oil and peanut oil, linalool appeared in the bloodstream within 5 min of the end of the massage (total massage time was 10 min), and the maximum concentration was observed after 19 min [43]. When administered orally, linalool appeared in the blood of the subjects approximately 10–15 min after consuming 200 μL of lavender oil, with the maximum concentration observed after 30 min [46].

**Scheme 1:**
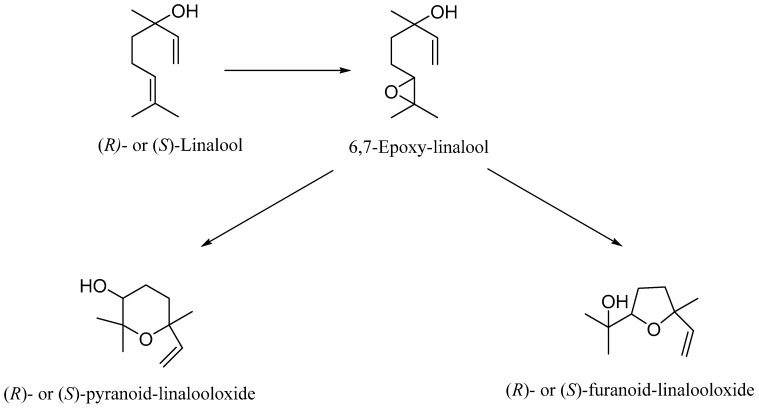
Biotransformation of linalool by CYP2D6.

Linalool biotransformations were performed using human recombinant CYP2D6 and CYP2C19. Both CYP isoforms are present in human skin. The major product was *cis*- and *trans*-8-hydroxylinalool. Allylic hydroxylation was catalyzed by both enzymes, while enzymatic epoxidation of linalool to yield 6,7-epoxy-linalool was catalyzed only by CYP2D6. Trace formation of (*R/S*)-furanoid-linalool oxide and (*R/S*)-pyranoid-linalool oxide, which are products of intramolecular rearrangement due to the attack of the hydroxyl group on the epoxy bond, has also been observed. It is worth noting that the enzymatic affinity of CYP2D6 for the enzymatic epoxidation of the double bond was approximately 3–10 times lower than the enzymatic affinity for the hydroxylation reaction. The catalytic yields of cyclic ether formation were approximately in the same range but approximately 3000–4000 times lower than that of the hydroxylation reaction [47] (Scheme 1).

**Scheme 2:**
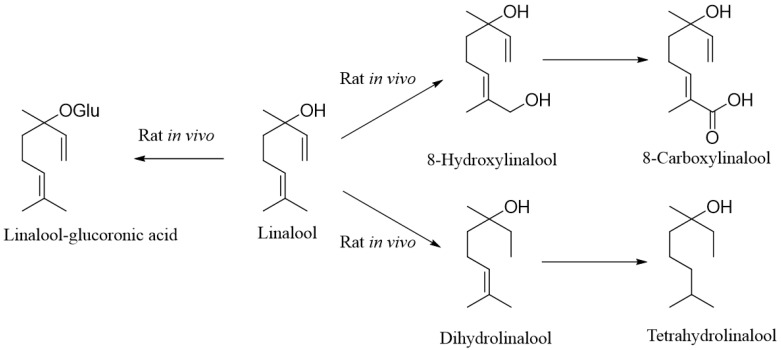
Linalool transformation observed in rat in vivo.

In animals, the first attempts to study the metabolism of linalool in vivo were made at the beginning of the 20th century, when in 1904, Hildebrandt conducted research on rabbits. He showed that linalool is excreted as glucuronide [48]. In 1974, Parke et al. [49] investigated the distribution of [^14^C] linalool in rats and observed that after a single oral dose, about 58% of the administered linalool was excreted in the urine, and 25% as ^14^CO_2_ in the expired air and 16% in the faeces after 72 h. About 10% of the administered dose was excreted as [^14^C] urea in the urine. Additionally, linalool derivatives such as dihydro- and tetrahydrolinalool (free and conjugated) have also been detected in significant amounts in urine. It was observed that when administered to rats, linalool induced hepatic microsomal cytochrome P-450 and UDP-glucuronyl transferase [50]. Further studies have shown that linalool is first converted by cytochrome P-450 (CYP) isoenzymes to dihydrolinalool and tetrahydrolinalool and to 8-hydroxylinalool, which is further oxidized to 8-carboxylinalool (Scheme 2). The resulting compound is then converted into a glucuronide conjugate and, as such, form excreted from the body [51]. It is worth mentioning that pulmonary microsomes obtained from rats pre-treated with *b*-naphthoflavone catalyzed the formation of 8-hydroxylinalool from linalool [52].

Further studies showed that in rats, (3*R*)-(−)-linalool induces CYP2A and does not affect 2B, 2C6, 2C11 and 3A [53]. The CYP2A family in rats includes CYP2A1 and CYP2A2 in liver tissue and CYP2A3 in the lung. This enzyme shows about 60% homology in the amino acid sequence with human CYP2A and is responsible in humans for 7α-hydroxylation of testosterone; activates several carcinogens, including nitrosamines and aflatoxins; and participates, among others, in the metabolism of nicotine or coumarin. However, it should be considered that in the above study, the doses of linalool were very high, so the researchers postulate that the effect of linalool on drug metabolizing enzymes is not clinically significant. Therefore, further studies on the actual metabolism of linalool using other experimental systems, such as human liver microsomes, are necessary [54].

## 3. Antimicrobial Activity

Linalool has a known antimicrobial effect against both bacteria and fungi. The list of strains against which it is active is summarized in Table 2. Although it is a component of several plants, i.e., coriander, rosewood, basil, mint, ginger, laurel, thyme, sage, nutmeg or cinnamon, it is poorly used in nutritional systems due to its physical properties: it is poorly soluble in water and has high volatility and low stability. However, due to several proven activities, linalool is a promising ingredient of food.

There are many reports in the literature on the antimicrobial properties of linalool; however, especially those that prove that this monoterpene is effective against life-threatening strains, including multi-drug resistant strains, i.e., *E. coli* O157:H7 [55] or *Pseudomonas aeruginosa* [56], deserve attention. In addition, the effective effect of linalool against bacteria associated primarily with gastrointestinal infections (e.g., *Salmonella enterica* subsp. *enterica* serovar Typhi, *Salmonella enterica* subsp. *enterica* serovar Typhimurium, *E. coli*, *Vibrio fluvialis*, *Campylobacter jejuni* and *Staphylococcus aureus*) [57,58,59], as well as with periodontal diseases and caries (e.g., *Porphyromonas gingivalis*, *Prevotella intermedia*, *Prevotella nigrescens*, *Fusobacterium nucleatum*, *Aggregatibacter actonomycetemcomitans*, *Streptococcus mutans* and *Streptococcus sobrinus*) [60] and diseases caused by opportunistic bacteria (*P. aeruginosa* and *Staphylococcus epidermidis*) [26,61]. Adding linalool to food would have broadly understood positive effects as it has been shown to be active against microbes contaminating the food: *Campylobacter* spp. (undercooked poultry, raw milk and contaminated water) and *Salmonella* spp. (eggs, meat, poultry, milk, and unwashed green fruit and vegetables)) as well as *Listeria monocytogenes* [62] (soft and mature cheeses, raw milk, cream, eggs, ice cream, meat, smoked fish, pork, sausages, raw vegetables and fruits, and frozen foods) and *E. coli* (unwashed vegetables and fruits, raw meat and water). However, the physical properties of linalool turn out to be a problem, mainly its solubility, stability and sensory properties, as well as its susceptibility to react with factors included in the food, i.e., water, fat, protein, salt and pH. This difficult to overcome barrier related to the bioavailability of this monoterpene in nutritional systems was solved by Soković et al. [61]. In their research, they proved that linalool can be closed in nanoemulsions, colloidal dispersions, which are characterized by high stability. Moreover, such a system shows an order of greater microbial activity against *S.* Typhimurium ATCC 13311 than pure monoterpene. This is most likely due to the fact that it penetrates better into the bacterial cell. However, despite this, such a system is less effective at reducing the structure of the biofilm. More effective in this case, due to its small size, is pure linalool (effectiveness: linalool nanoparticle; 64.5–56.9%). To increase the antimicrobial activity, to eliminate negative organoleptic effects and to change the natural properties of food after adding the desired concentrations of linalool, it was combined with food ingredients acceptable for consumption, including vitamin C and copper (II) ions. This treatment significantly reduced the MIC values to 32 mM, 7.5 mM and 212 µM for *V. fluvialis*, *S. enterica* subsp. *enterica* serovar Typhi and *E. coli* ATCC 25922, respectively [57]. A synergistic effect of these compounds was also observed in this combination. A synergistic effect of these compounds was also observed in this combination.

Linalool as a component of many essential oils (EO) is used not only in cosmetology but also in medicine. It has been shown that its addition to EO, i.e., *Thymus vulgaris* (thyme), *Juniperus communis* (juniper), *Pelargonium graveolens* (geranium), *Citrus bergamia* (bergamot), *Citrus paradise* (grapefruit), *Lavandula angustifolia* (lavender), *Cinnamomum zeylanicum* (cinnamon), *Melaleuca alternifolia* (tea tree) and *Syzygium aromatum* (clove) increase antimicrobial activity against both Gram-positive bacteria (*S. aureus*, *L. monocytogenes*) and fungi (*Candida albicans*, *Aspergillus brasiliensis*), and Gram-negative bacteria (*E. coli, P. aeruginosa, S.* Typhimurium), the treatment of which is very often problematic because they are characterized by a high level of resistance to antibiotics used in therapy [63,64]. In addition, it has been proven that plants in which linalool constitutes a high percentage of the entire composition, i.e., *Lavandula angustifolia*, La (27.2%) and *Ocimum basilicum*, Ob (69.3%), exhibit strong antimicrobial activity against Gram-positive strains: *Bacillus subtilis* (MIC = 4 µg/mL), *S. epidermidis* (MIC = 4 µg/mL) and *S. aureus* (MIC = 5 µg/mL for La and 4.5 µg/mL for Ob) [62].

Linalool also proved to be promising in combating infectious diseases not only in humans but also in, e.g., fish. This monoterpene has been shown to be very effective against *Shewanella putrefaciens* (MIC = 1.5 µg/mL), a pathogen that poses a high risk to fish and seafood [65].

In addition to the action on bacterial pathogens, linalool also exhibits antifungal properties, both against yeast-like fungi, e.g., *C. albicans* [66], and dermatophytes, e.g., *Trichophyton rubrum*. De Oliveira Lima et al. [67] demonstrated a positive effect (MIC = 256–512 µg/mL) of linalool on *T. rubrum* strain resistant to fluconazole, commonly used in the treatment of dermatophytosis.

Recent antifungal research shows great promise in the treatment of diseases caused by *Aspergillus flavus*. Li et al. [68] proved the complete inhibition of the growth of this fungus after contact with gaseous (0.571 µg/mL) and liquid (1.2 µg/mL) linalool. While the study looks at infected wheat grains, it is nonetheless an interesting alternative for treating pulmonary aspergillosis caused by this aspergillosis.

It is also interesting that there are already natural preparations on the market with proven antifungal properties that contain linalool, incl. *Ocimum sanctum* [69] and *Ocimum basilicum* [70] used in the form of an infusion, powdered herb, or tincture with the addition of honey.

In the publications we cited for antimicrobial activity, the essential oils whose main component is linalool was used for the study. In terms of biological properties, including participation in metabolic pathways, the molecule of linalool from natural sources or by organic synthesis shown no differences.

### 3.1. Mechanism of the Antibacterial Action of Linalool

Research described in the literature has shown that linalool can disrupt cellular function and even cell death. This monoterpene, after contact with a bacterial cell, first acts on the cell membrane, causing damage to its function by reducing the membrane potential (MP) and structure, causing intracellular leakage of macromolecules (DNA, RNA and proteins). In addition, it inhibits the synthesis of pathways related to obtaining energy by bacteria. It also leads to metabolic dysfunctions and inhibits the activity of key enzymes, causing an abnormal structure of the bacterial cell and thus leading to its death [58,65,71,72].

#### 3.1.1. Alteration of Amino Acid Metabolism

Bacterial cells exposed to stress from the external environment usually defend themselves by activating some pathways of amino acid biosynthesis (Table 3). Such behavior enables them to survive. Guo et al. [65] showed that in the presence of linalool in a bacterial cell, there is a decrease in the cellular activity of alkaline phosphatase (AKP), which in turn leads to the destruction of the integrity of the cell wall.

Moreover, it has been proven that this monoterpene contributes to an increase in the concentration of histidine and methionine. These amino acids are involved in the structural phenotype of the bacterial biofilm. Thus, linalool directly contributes to the damage of the biofilm structure, i.e., the physical barrier that effectively protects microorganisms against the action of antibiotics, antiseptics and the host’s immune system [58].

Linalool also contributes to disturbing the pH homeostasis of the bacterial cell by increasing the concentration of lysine while reducing the concentration of L-aspartate. Both of these amino acids, apart from L-glutamate, L-glutamine and L-arginine, play a huge role in the homeostatic mechanisms that determine the proper functioning of the body in prokaryotes [65].

#### 3.1.2. Change in Carbohydrate Metabolism

Under various conditions of environmental stress, microorganisms generally reduce their energy metabolism, aiming to save energy for the longest possible period of time (Table 4). Glycolysis, the tricarboxylic acid (TCA) cycle and the electron transfer chain are keyways of providing the energy needed for bacterial cell activity. The influence of linalool on the necessary substances involved in the proper acquisition of energy by the bacterial cell, i.e., pyruvate, citrate, fumarate, lactate and 2-oxoglutarate, has been shown. Lowering their concentration affects the TCA cycle and glycolysis and contributes to the disruption of ATPase, ultimately leading to the disruption of energy production. Under such conditions, the death of the bacterial cell occurs [71].

In addition, linalool has been shown to reduce the activity of pyruvate kinase (PK) and succinate dehydrogenase (SDH), enzymes that play a key role in the TCA cycle. When their activity is disturbed, the production of pyruvate and ATP (interference with the starting point of the TCA cycle) and the second mitochondrial complex of the electron transport chain are disrupted (the correct conversion of energy from nucleotides into ATP energy) [65].

#### 3.1.3. Changes in Lipid Metabolism

Bacteria are not indifferent to environmental stress. They respond to it by changing the fluidity of the cell membrane through changes in the ratio of C16 and C18 fatty acids and the ratio of saturated and unsaturated fatty acids (Table 5).

High levels of saturated fatty acids, i.e., linolenic, linoleic, oleic, arachidonic, myristic, octadecanedioic and palmitic acids, indicate low fluidity of the cell membrane. In the presence of linalool, the content of C16 and C18 fatty acids as well as saturated and unsaturated fatty acids increases. In addition, this monoterpene shows the ability to bind to phospholipids, thus damaging the structure of the membrane, because phosphadylcholine (PC) and phosphatidylethanolamine (PE), the main phospholipids present in the cell membrane, are responsible for the integrity and fluidity of the bacterial cell [72].

### 3.2. Antifungal Properties of Linalool

In the pathogenic process of fungi, their adhesion to host tissues, germination of conidia, and the formation of hyphae as well as penetration into the cell are important. De Oliveira Lima et al. [59,67] showed that linalool has the ability to disrupt the morphogenesis of the fungus, thereby preventing the formation of dermatophyte mycelium, which ultimately leads to inhibition of fungal growth at the site of infection and thus blocking further stages of infection. In addition, previous studies have already proven that linalool inhibits the process of conidiogenesis and conidia germination and disrupts the proper formation of chlamydoconidia, hyphae and the so-called “germ tubes” [73].

A key enzyme in the biosynthesis of ergosterol, a sterol that is an integral component of the fungal cell wall involved in the modulation of electrolyte flow, is 3-hydroxy-3-methylglutaryl-coenzyme A (HMG-CoA) reductase. It oversees the conversion of HMG-CoA to mevalonic acid. Mate et al. [66] showed that linalool directly influences the composition of the fungal plasma membrane by inhibiting the expression of HMG-CoA reductase. Moreover, these authors proved that linalool induces changes in the fatty acid composition in the fungal cell by increasing the level of polyunsaturated and unsaturated fatty acids. On the other hand, the cytotoxicity of this monoterpene on the fungal cell is a consequence of the accumulation of reactive oxygen species (ROS), including superoxide •O_2_^−^, peroxide H_2_O_2_ and hydroxyl •OH.

Research shows that linalool, by directly or indirectly influencing the integrity and function of the plasma membrane in fungal cells, can inhibit their growth and induce significant morphological changes (Table 6).

## 4. Microbial Biotransformation of Linalool

Antibiotics are commonly used in the treatment of various infections and, when enter surface waters and soil, negatively affect the local ecosystems. For this reason, it is important to identify the possible degradation pathways for any new antimicrobial drug, including linalool. The first stage of degradation is often biotransformation processes, which are discussed in detail in this section.

One of the main reactions that linalool undergoes under the influence of many microorganisms is epoxidation of the double bond followed by cyclization to obtain cyclic ethers. In the case of the (3*R*)-(−)-isomer of linalool, the products can be *trans-*(2*R*,5*R*)-furanoid linalool oxide, *cis*-(2*R*,5*S*)-furanoid linalool oxide, and *trans*-(2*R*,5*S*)-pyranoid linalool oxide and *cis*-(2*R*,5*R*)-pyranoid linalol oxide. On the other hand, as a result of cyclization of the (3*S*)-(+)-isomer of linalool, the products may be *trans*-(2*S*,5*S*)-furanoid linalool oxide, *cis*-(2*S*,5*R*)-furanoid linalool oxide, *trans*-(2*S*,5*R*)-pyranoid linalool oxide and *cis*-(2*S*,5*S*)-pyranoid linalool oxide [76].

Both racemic (±)-linalool and (3*R*)-(−)-linalool were used as substrate for biotransformation by *Aspergillus niger* ATCC 9142. Biotransformation was carried out for 20 days. At the time of inoculation, 50 µL of linalool was added to 100 mL of the medium; after 14 days, another 100 µL of linalool was added; and after 20 days, the transformation process was completed. In both cases, epoxidation of the double bond took place, followed by cyclization of the resulting epoxide. In the case of (±)-linalool, a mixture of *cis*-(2*S*,5*R*)- and *trans*-(2*R*,5*R*)-furanoid linalool oxide (15–24% yield), and a mixture of *cis*-(2*S*,5*S*)- and *trans*-(2*R*,5*S*)-pyranoid linalool oxide (5–9% yield) resulted. In turn, (3*R*)-(−)-linalool was transformed mainly into *trans*-(2*R*,5*R*)-furanoid linalool oxide with ee > 95% and *trans*-(2*R*,5*S*)-pyranoid linalool oxide [77].

Another strain: *A. niger* DSM 821 was used to biotransform (3*S*)-(+)-linalool with 77% purity. The main products were *cis*-(2*S*,5*R*)-furanoid linalool oxide with a yield of 30% and an enantiomeric excess of 100%, and *cis*-(2*S*,5*S*)-pyranoid linalool oxide obtained with a yield of 14%. Moreover, it was found that the (3*S*)-(+)-isomer of linalool was transformed much better than the (3*R*)-(−)-isomer of linalool, which did not exceed 4% conversion [78].

The same cyclic products were formed during the biotransformation of (3*R*)-(−)-linalool by the *Diplodia gossypina* ATCC 10936 fungal strain. In this case, 10 μL of linalool was added to 100 mL of the grown culture and the transformation process was carried out for 24 h. As a result, *trans*-(2*R*,5*S*)-furanoid linalool oxide with an efficiency of 12% and ee = 97% and *trans*-(2*R*,5*R*)-pyranoid linalool oxide with an efficiency of 21% and ee = 98% were obtained [79].

Studies using the *Corynespora cassiicola* DSM 62475 strain to transform both the racemic mixture of linalool and the pure (3*R*)-(−)-enantiomer have shown that the formation of cyclic oxygen derivatives of linalool is highly stereospecific. The products obtained from (±)-linalool were *trans*-(2*R*,5*R*) and *cis*-(2*S*,5*R*)-furanoid linalool oxide and *trans*-(2*R*,5*S*) and *cis*-(2*S*,5*S*)-pyranoid linalool oxide. For the (3*R*)-(−)-enantiomer of linalool, the main products were *trans*-(2*R*,5*R*)-furanoid linalool oxide and *trans*-(2*R*,5*S*)-pyranoid linalool oxide [80].

In another experiment, *Colletotrichum acutatum* TQ058A and *C. nymphaeae* CBMAI 0864 strains were used for the biotransformation of linalool. Both strains converted linalool to *cis*- and *trans*-furanoid linalool oxide. In the case of the *C. acutatum* strain, the products were formed in the amount of 0.04 g/L and 0.03 g/L, respectively. In the case of the *C. nymphaeae* strain, these values were slightly higher and amounted to 0.05 g/L and 0.04 g/L, respectively was obtained [81].

Not only fungi but also microalgae can convert linalool into cyclic ethers. As a result of a 24 h biotransformation using the microalgae *Oocystis pusilla*, (3*R*)-(−)-linalool was transformed into a mixture of *trans*-(2*R*,5*R*)-furanoid linalool oxide with an efficiency of 8% and *trans*-(2*R*,5*S*)-pyranoid linalool oxide with an efficiency of 6% [82].

**Scheme 3:**
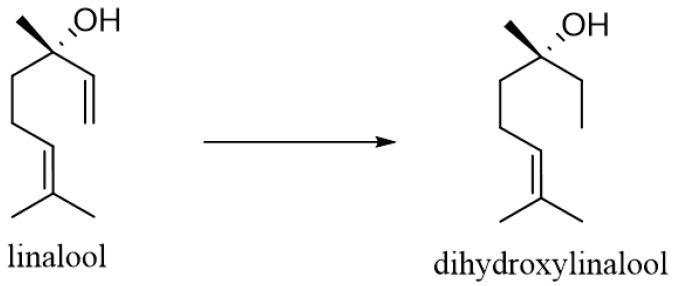
Biotransformation of linalool by *Chlorella* spp.

On the other hand, when (3*S*)-(+)-linalool was used as a substrate for biotransformation, it turned out that microalgae led to not only double bond epoxidation and subsequent cyclization of linalool but also a reduction in double bonds. Microalgae isolated from rice fields in Iran were immobilized in calcium alginate and incubated with substrate for 24 h. The *Chlorella* spp. MCCS 028 strain transformed the substrate into a mixture of *cis*-(2*S*,5*R*)- and *trans*-(2*S*,5*S*)-furanoid linalool oxide (both with a yield of 1.4%) and into dihydrolinalool with a yield of 3.2%. On the other hand, the strains of *Chlorella* spp. MCCS 029 and *Chlamydomonas* spp. MCCS 036 were only able to reduce linalool to dihydrolinalool, which allowed for 4.1% and 3.3% of this compound to be obtained, respectively [83] (Scheme 3).

Some of the tested microorganisms proved to be able to convert linalool into other monoterpenoids.

**Scheme 4:**
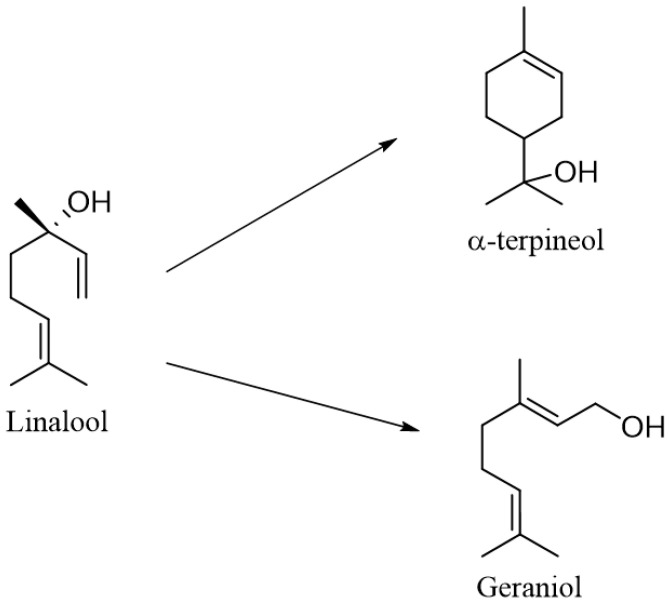
Biotransformation of linalool by fungus from Brazilian fruits.

Fungi isolated from fruits found in Brazil were used for the biotransformation of (3*S*)-(+)-linalool (Scheme 4). These strains were incubated in YM medium for 72 h, and 0.1% of the substrate was added. The substrate addition was continued every 24 h for the next 3 days. After another 24 h, extraction was performed. One strain, designated LB-2002, was able to convert the substrate to *cis*-(2*S*,5*R*)- and *trans*-(2*S*,5*S*)-furanoid linalool oxide, with efficiencies of 65% and 35%, respectively. For the first of these products, the yield was 160 mg/L. Similar products were obtained using the LB-2008 strain isolated from acerola for biotransformation. The LB-2077 strain, isolated from graviola, converted linalool to geraniol with a yield of 7 mg/L. The LB-2010 and LB-2050 strains, on the other hand, were able to convert linalool to α-terpineol with capacities of 150 and 130 mg/L, respectively [84].

The yeast *Saccharomyces cerevisiae* IWD72 (prototrophic haploid, MATa), *Torulaspora delbrueckii* NCYC 696 and *Kluyveromyces lactis* IFO1267 were used to biotransform the racemic mixture of (±)-linalool. Twenty-five µg/mL of linalool was added to the grown cultures, and the biotransformations were carried out for 72 h. The only product of biotransformation was (±)-α-terpineol. It was obtained with yields of 12.2% for the *S. cerevisiae strain* and 20.8% for the *T. delbrueckii* strain. The *K. lactis* strain, which gave 31.4% of α-terpineol, turned out to be the most effective [85].

**Scheme 5:**
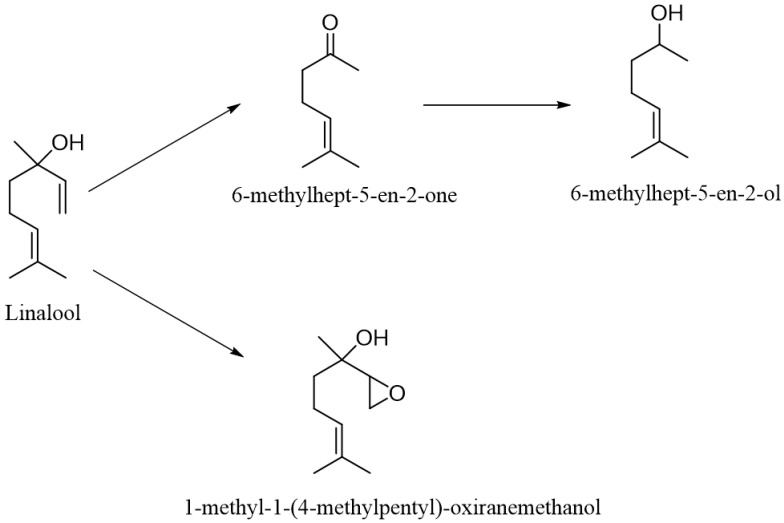
Biotransformation of linalool by *Fusarium* strains from citrus.

In turn, *Fusarium* strains isolated from the fruits of the genus *Citrus* growing in the Amazon rainforest were also used for the biotransformation of (±)-linalool. After adding the substrate in the amount of 1 g/L to the grown culture, the transformation was carried out for 5 days. Different products were obtained depending on the strain used. The *Fusarium* spp. 1D2 strain transformed linalool via epoxy into a mixture of *cis*- and *trans*-furanoid linalool oxide (yield 39 and 37%, respectively). As a result of using the *Fusarium* spp. 1C5 strain, *cis*-furanoid linalool oxide was obtained with a yield of 32% and a mixture of *cis*- and *trans*-pyranoid linalool oxide (yield of 12% and 2%, respectively). The *Fusarium* spp. 1DC5 and *F. concentricum* strains were able to epoxidate the terminal double bond in the linalool molecule to give 1-methyl-1-(4-methylpentyl)-oxiranemethanol with a yield of 42%. The *F. fujikuroi* strain was able to cleave the vinyl fragment from the linalool molecule, converting it into 6-methylhept-5-en-2-one with a yield of 49%. The second product (in the amount of 10%) was also observed in the post-reaction mixture, resulting from the reduction in the carbonyl group, i.e., 6-methylhept-5-en-2-ol [86] (Scheme 5).

A key reaction in the metabolism of linalool observed in animals, plants and microorganisms is the C-8 carbon hydroxylation reaction. In the case of microorganisms, this reaction can additionally be an intermediate step in the formation of cyclic ethers.

Such mixtures of various compounds that are both cyclic ethers and a hydroxyl derivative of linalool have been observed during the biotransformation of racemic (±)-linalool. In this case, the substrate was biotransformed in cultures of *A. niger* ATCC 16404 (for 9 days), *A. niger* DSM 821 (for 6 days), *Botrytis cinerea* 5901/2 (for 9 days), *B. cinerea* 02/FBII/2.1 (for 3 days) and *C. cassiicola* DSM 62475 (for 3 days). Three of them—*A. niger* DSM 821, *B. cinerea* 02/ FBII/2.1 and *C. cassiicola* DSM 62475—transformed linalool into a mixture of *trans*-(2*R*,5*R*)- and *cis*-(2*S*,5*R*)-furanoid linalool oxide at efficiencies of 7.7, 24.9 and 81.6%, respectively, and a mixture of *trans*-(2*R*,5*S*)- and *cis*-(2*S*,5*S*)-pyranoid linalool oxide at efficiencies of 17.4, 8.6 and 14.9%, respectively. In addition to the above-mentioned products, *A. niger* DSM 821, *B. cinerea* 02/FBII/2.1 strains carried out the hydroxylation of linalool to 8-hydroxylinalool with yields of 14.2 and 17.2%, respectively. In the case of *A. niger* ATCC 16404 strain, only *cis*-(2*S*,5*R*)-furanoid linalool oxide was observed with a yield of 5.3% and 8-hydroxylinalool was observed with a yield of 21%. On the other hand, the *B. cinerea* 5901/2 strain was unable to convert linalool to cyclic ethers, but it only carried out the substrate hydroxylation reaction to 8-hydroxylinalool with an efficiency of 60% [76].

8-hydroxylinalool was also obtained as a result of the biotransformation of (±)-linalool by the enzyme CYP111A2 isolated from *Novosphingobium aromaticivorans* [87].

**Scheme 6:**
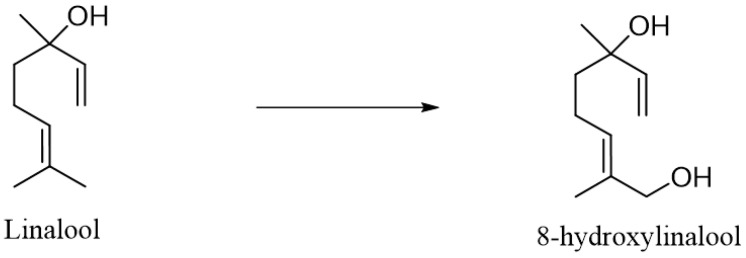
Biotransformation of linalool by *B. cinerea* strains.

*B. cinerea* strains (5901/2, 5909/1 and 5899/4), which were grown on grape must of the Milller-Thurgau variety, were also used for the biotransformation of (±)-linalool. The flasks containing the sterilized must were inoculated with the microorganism and incubated at 25 °C for 2 weeks. Linalool was added to the culture prepared in this way. The major product of the biotransformation (greater than 90%) was found to be 8-hydroxylinalool [88] (Scheme 6).

The same derivative of linalool was also obtained when *Nicotiana tabacum* “Bright Yellow” tissue culture was used for the biotransformation of linalool. Callus tissues obtained from the plant stem were pre-cultured for 3–4 weeks in medium containing 2 ppm 2,4-dichlorophenoxyacetic acid and 2% sucrose. (3*R*)-(−)-Linalool (20 mg per flask) was added to the grown culture (40–60 g per flask) and then shaken at 25 °C in the dark for 7 days. 8-Hydroxylinalool was obtained in a yield of 16.5% [89].

**Scheme 7:**
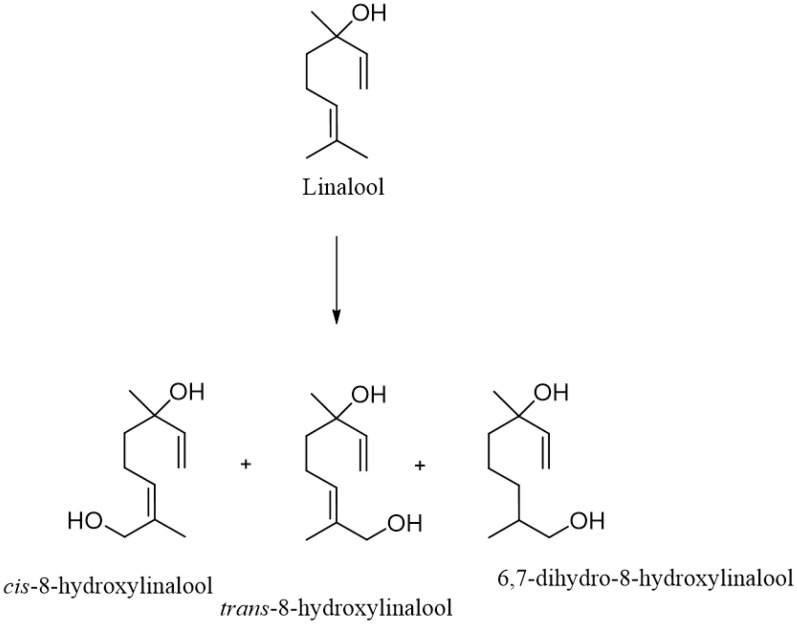
Biotransformation of linalool by a suspension culture of young fruits of *Vitis vinifera* L. cv. Gamay Freau.

In another experiment, a suspension culture was used for the biotransformation of linalool, which was derived from fragments of young fruits of *Vitis vinifera* L. cv. Gamay Freau (Scheme 7). The process was carried out for 48 h. Linalool was transformed into three hydroxy derivatives, *cis*- and *trans*-8-hydroxylinalool and 6,7-dihydro-8-hydroxylinalool. In the latter case, in addition to the hydroxylation of the C-8 carbon, a reduction in the vinyl double bond took place [90].

## 5. Conclusions

Linalool is one of the most important fragrance compounds, as the market for the production of this terpenoid alcohol was estimated to be around USD 10 billion in 2019. It is commonly found in food and used in the production of perfumes, lotions, soaps and shampoos, detergents and cleaning products. An interesting innovation is the possibility of using linalool as an ingredient in active packaging also used in food production.

Linalool is mainly obtained by chemical synthesis. However, increasing awareness of climate change, environmental pollution generated by the chemical industry is prompting the search for alternative methods of obtaining this compound. Linalool is a component of many essential oils and can be isolated from them, e.g., by fractional distillation. However, over-harvesting of plants with valuable essential oils can also be associated with negative environmental impacts. Therefore, in order to maintain the sustainability trend in the industry, efficient biotechnological methods for the production of linalool using genetically transformed microorganisms should be sought. Unfortunately, the instability of DNA pathway constructs and the maintenance of a fully functioning metabolic pathway in microorganisms is still a major problem; therefore, the methods developed so far have not gone beyond the laboratory scale. It is worth noting that in fermentation processes, in order to reduce costs, attempts are also being made to use food industry residues or by-products as substrates.

Linalool has a number of biological activities as it has antioxidant and anti-inflammatory, antimicrobial, cardioprotective, neuroprotective, sedative, anxiolytic, anticonvulsant and anticancer effects. In terms of its antibacterial activity, it is active mainly against Gram-negative bacteria, as shown in Table 2. It should be remembered that these bacteria, along with Gram-positive bacteria of *Staphylococcus* spp. and *Enterococcus* spp., are the main etiological factor of nosocomial infections, i.e., infections that cause a huge problem in medicine all over the world. The main problems that may arise when treating infections caused by Gram-negative bacteria are build-up of resistance to the antibiotics used, the increasing proportion of bacteria with a broad panel of resistance (i.e., non-fermenting rods) in infections, the ability to persist in a hospital environment for long periods and chronic infections. Due to the fact that more and more often in the treatment of these infections, even the so-called last chance antibiotics, linalool seems to be a promising future. Its mechanism of action on microbes is multifaceted, which is important in combating superbugs that mutate at a rapid pace. Therefore, it is worth considering the use of combination therapy—administration of an antibiotic with a natural compound, which is linalool, in the case of synergistic action of both compounds. Such an approach may reduce the emergence of drug resistance to antibiotics of “last resort” of microorganisms previously sensitive to them. Therefore, it is puzzling that still little is known about the interactions of linalool with known drugs or with food ingredients. First of all, there are no unequivocal results of research on whether and to what extent linalool acts as an inducer for all CYP isoforms occurring in humans. Therefore, further research is needed in this area using such modern methods as transcriptomics and proteomics techniques, metabolomics, X-ray crystallography, spectroscopy and computer modeling. Clinical trials of the combination of antibiotics with linalool are also necessary, with a particular emphasis on severely ill patients, when factors such as organ failure, increased vascular permeability and renal replacement therapies may alter the pharmacokinetics of any medication used, which may significantly reduce the effectiveness of the treatment undertaken.

A somewhat neglected area of research is the biotransformation of linalool. Finding strains of microorganisms capable of transforming compounds with antimicrobial activity is extremely tedious and laborious, which may explain the limited number of publications on this subject. However, research of this type is of particular importance in the context of a holistic view of the functioning of linalool in the environment.

## Figures and Tables

**Table 1:** Plant resources of linalool.

Plant	Plant Part	Percentage [%]	Ref.
*Aniba rosaeodora* Ducke	Wood	100	[7]
*Cinnamomum camphora* Nees & Eberm var. *linaloolifera*	Leaf	95	[8]
*Mentha spicata*	Leaf	94	[9]
*Cinnamomum osmophloeum* ct.	Leaf	94	[10]
*Cinnamomum camphora* Nees & Eberm var. *linaloolifera*	Wood	90	[11]
*Coriandrum sativum* L.	Seeds	83	[12]
*Orthodon linalooliferum* Fujita	Aerial parts	82	[13]
*Coriandrum sativum* L.	Mericarp	79	[14]
*Zanthoxylum alatum* Roxb	Seeds	71	[15]
*Coriandrum sativum* L.	Seeds	66	[16]
*Lavandula angustifolia*	Whole plant	54	[17]
*Lippia alba*	Leaves	53	[18]
*Hyssopus officinalis*	Aerial parts	51	[19]
*Lavandula latifolia*	Whole plant	51	[14]

**Table 2:** Antimicrobial activity of linalool against bacteria.

Strain	Activity	Reference
Gram-Positive Bacteria		
*Micrococcus flavus* ATCC 9341	4 µg/mL	[61]
*Bacillus subtilis* ATCC 10707	4 µg/mL	[61]
*Staphylococcus epidermidis* ATCC 12228	4 µg/mL	[61]
*Staphylococcus aureus* ATCC 25923	5 µg/mL	[61]
*Staphylococcus aureus* NCTC 10788	13 mm	[63]
*Listeria monocytogenes*	5 µg/mL	[61]
*Listeria innocua* NRRLB-33314	1%	[55]
Gram-Negative Bacteria		
*Salmonella enterica* subsp. *enterica* serovar Enteritidis ATCC 13076	5 µg/mL	[61]
*Salmonella enterica* subsp. *enterica* serovar Typhimurium ATCC 13311	5 µg/mL	[61]
*Salmonella enterica* subsp. *enterica* serovar Typhimurium CCM 5445	0.7%	[55]
*Vibrio fluvialis* L-15318	32 mM	[57]
*Salmonella enterica* subsp. *enterica* serovar Typhi ATCC 14901	7.5 mM	[57]
*E. coli* ATCC 25922	212 µM	[57]
*E. coli* NCTC 12923	21 mm	[63]
*E. coli* ATCC O157:H7	6 µg/mL (0.6%)	[55,61]
*Pseudomonas aeruginosa* NCTC 12924	8 mm	[63]
*Pseudomonas aeruginosa* ATCC 27853	7 µg/mL	[61]
*Pseudomonas aeruginosa* ATCC 9027	431 µg/mL	[56]
*Pseudomonas fluorescens* ATCC 13525	1.25 µg/mL	[64]
*Enterobacter cloacae* (h.i.)	6 µg/mL	[61]
*Proteus mirabilis* (h.i.)	6 µg/mL	[61]
*Campylobacter jejuni* ATCC 33560	0.5 µL/mL	[59]
*Campylobacter coli* ATCC 33559	0.5 µL/mL	[59]
*Serratia liquefaciens* NRRLB-951	1%	[55]
*Porphyromonas gingivalis* ATCC 33277	0.8 mg/mL	[60]
*Porphyromonas gingivalis* ATCC 49417, ATCC 53978	0.1 mg/mL	[60]
*Prevotella intermedia* ATCC 25611	0.2 mg/mL	[60]
*Prevotella intermedia* ATCC 49046	1.6 mg/mL	[60]
*Prevotella nigrescens* ATCC 25261, ATCC 33563	0.8 mg/mL	[60]
*Fusobacterium nucleatum* subsp. *nucletum* ATCC 25586	0.2 mg/mL	[60]
*Fusobacterium nucleatum* subsp. *polymorphum* ATCC 10953	0.2 mg/mL	[60]
*Fusobacterium nucleatum* subsp. *vincenti* ATCC 49046	0.1 mg/mL	[60]
*Fusobacterium nucleatum* subsp. *fusiforme* ATCC 51190	0.2 mg/mL	[60]
*Fusobacterium nucleatum* subsp. *animals* ATCC 51191	0.2 mg/mL	[60]
*Aggregatibacter actinomycetemcomitans* ATCC 33384, ATCC 43717, ATCC 43718	0.1 mg/mL	[60]

Legend: h.i.—human isolate.

**Table 3:** Effect of linalool on amino acid metabolism.

Parameter	Linalool	Effect
Alkaline phosphatase (ā)	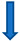	Destruction of the integrity of the cell wall
Histidine and methionine	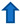	Damage to the structure of the biofilm
L-glutamate, L-glutamine, L-arginine, L-aspartate	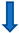	Disturbance of the pH homeostasis of the bacterial cell

Legend: ā—enzyme activity.

**Table 4:** Effect of linalool on carbohydrate metabolism.

Parameter	linalool	Effect
Pyruvate, Citrate, Fumarate, Lactate, 2-Okoglutarate	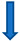	ATPase disturbance ≥ disturbance of energy production ≥ abnormal TCA cycle and glycolysis
Pyruvate kinase (ā)	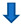	Disrupted TCA cycle
Succinate dehydrogenase (ā)	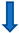	Disturbance of oxidative phosphorylation

Legend: ā—enzyme activity.

**Table 5:** Effect of linalool on lipid metabolism.

Parameter	Linalool	Effect
fatty acids: linolenic, linoleic, oleic, arachidonic, myristic, octadecadiene, palmitic	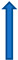	Low fluidity of the cell membrane
Phosphatidylcholine, phosphatidylethanolamine	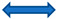 binding	Damage to the structure of the cell membrane

**Table 6:** Antimicrobial activity of linalool against fungi.

Strain	Activity of Linalool	Referece
*Candida albicans* NCPF3179	12 mm	[63]
*Candida albicans* ATCC 14053	8 mM	[73]
*Candida glabrata* (c.s.)	16 mM	[73]
*Candida tropicalis* ATCC 13803	125 µg/mL	[74]
*Candida tropicalis* (c.s.)	16 mM	[73]
*Trichophyton rubrum* LM 305	256 µg/mL	[67]
*Microsporum canis* (c.s.—LM910, LM82, LM29)	128 µg/mL	[75]
*Microsporum gypseum* (c.s.—LM512, LM130)	128 µg/mL	[75]

Legend: c.s.—clinical strain.

## Data Availability

Not applicable.

## Sample Availability

Samples of the compounds are not available from the authors.
